# 2,2′-Biimidazolium 5-amino-2,4,6-tri­bromo­isophthalate

**DOI:** 10.1107/S1600536810041899

**Published:** 2010-10-23

**Authors:** Kou-Lin Zhang, Han Huang, Seik Weng Ng

**Affiliations:** a College of Chemistry and Chemical Engineering, Yangzhou University, Yangzhou 225002, People’s Republic of China; b Department of Chemistry, University of Malaya, 50603 Kuala Lumpur, Malaysia

## Abstract

In the cation of the title salt, C_6_H_8_N_4_^2+^·C_8_H_2_Br_3_NO_4_^2−^, the dihedral angle between the two five-membered rings is 2.1 (3)°. In the anion, the mean planes of the carboxyl units are twisted from the benzene ring by 84.3 (4) and 86.2 (3)°. In the crystal, the components are linked by imidazolium–carboxyl­ate N—H⋯O hydrogen bonds, generating a chain running along [1
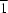
0].

## Related literature

For the structure of 5-amino-2,4,6-tribromidoisophthalic acid, see: Beck *et al.* (2009 ▶). For the structures of other 2,2′-bis­(imid­azolium) carboxyl­ates, see: Gao *et al.* (2009 ▶); Li & Yang (2007 ▶); Zhou *et al.* (2009 ▶).
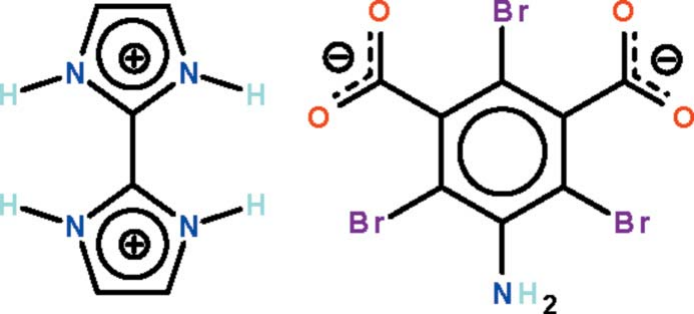


## Experimental

### 

#### Crystal data

C_6_H_8_N_4_^2+^·C_8_H_2_Br_3_NO_4_^2−^
*M* *_r_* = 552.00
Triclinic, 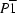

*a* = 9.0525 (10) Å
*b* = 9.2043 (10) Å
*c* = 11.5252 (12) Å
α = 90.262 (1)°
β = 108.332 (1)°
γ = 93.136 (1)°
*V* = 909.96 (17) Å^3^
*Z* = 2
Mo *K*α radiation
μ = 6.68 mm^−1^
*T* = 293 K
0.35 × 0.25 × 0.15 mm

#### Data collection

Bruker SMART APEX diffractometer
Absorption correction: multi-scan (*SADABS*; Sheldrick, 1996 ▶) *T*_min_ = 0.203, *T*_max_ = 0.434
8042 measured reflections
4104 independent reflections
3129 reflections with *I* > 2σ(*I*)
*R*_int_ = 0.025

#### Refinement

*R*[*F* ^2^ > 2σ(*F* ^2^)] = 0.035
*wR*(*F* ^2^) = 0.120
*S* = 1.12
4104 reflections
259 parameters
6 restraints
H atoms treated by a mixture of independent and constrained refinement
Δρ_max_ = 0.81 e Å^−3^
Δρ_min_ = −0.56 e Å^−3^

### 

Data collection: *APEX2* (Bruker, 2005 ▶); cell refinement: *SAINT* (Bruker, 2005 ▶); data reduction: *SAINT*; program(s) used to solve structure: *SHELXS97* (Sheldrick, 2008 ▶); program(s) used to refine structure: *SHELXL97* (Sheldrick, 2008 ▶); molecular graphics: *X-SEED* (Barbour, 2001 ▶); software used to prepare material for publication: *publCIF* (Westrip, 2010 ▶).

## Supplementary Material

Crystal structure: contains datablocks global, I. DOI: 10.1107/S1600536810041899/lh5152sup1.cif

Structure factors: contains datablocks I. DOI: 10.1107/S1600536810041899/lh5152Isup2.hkl

Additional supplementary materials:  crystallographic information; 3D view; checkCIF report

## Figures and Tables

**Table 1:** Hydrogen-bond geometry (Å, °)

*D*—H⋯*A*	*D*—H	H⋯*A*	*D*⋯*A*	*D*—H⋯*A*
N2—H2⋯O1	0.88 (1)	1.74 (2)	2.608 (4)	168 (6)
N3—H3⋯O3^i^	0.88 (1)	1.78 (2)	2.624 (5)	160 (5)
N4—H4⋯O4^i^	0.88 (1)	1.74 (1)	2.614 (5)	175 (7)
N5—H5⋯O2	0.88 (1)	1.79 (2)	2.636 (4)	160 (4)

Symmetry code: (i) 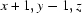.

**Fig. 1.**
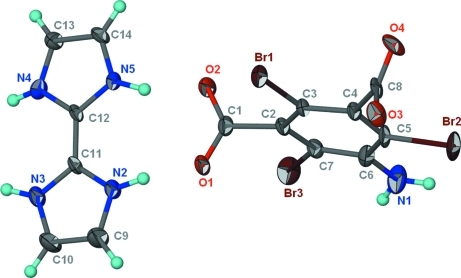
Thermal ellipsoid plot (Barbour, 2001) of C6H8N42+ C8H2NO4Br32– at the 50% probability level; hydrogen atoms are drawn as spheres of arbitrary radius.

**Fig. 2.**
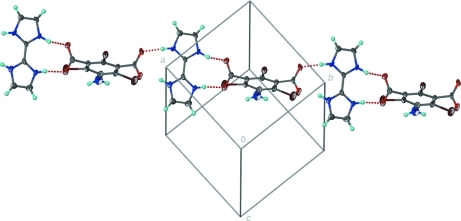
Part of the crystal structure showing hydrogen bonds as dashed lines.

## supplementary crystallographic information

### Comment

The crystal structure of 5-Amino-2,4,6-tribromoiodoisophthalic acid exists as a chains in which adjacent molecules are linked by O–H···O hydrogen bonds. In addition, pairs of chains are connected by further O–H···O hydrogen bonds (Beck *et al.*, 2009). This acid furnishes a small number of coordination compounds. An attempt to synthesize a lead(II) derivative that can be linked by 2,2'-biimidazole gave instead the title salt, [C_6_H_8_N_4_]^2+^ [C_8_H_2_NO_4_I_3_]^2–^(1). Other examples of crystal structure of 2,2'-bis(imidazolium) carboxylates already appear in the literature (Gao *et al.*, 2009; Li & Yang, 2007; Zhou *et al.*, 2009).

The asymmetric unit of (1) is shown in Fig. 1. The cation is nearly planar as its two five-membered rings are twisted along the C_imidazolyl_–C_imidazolyl_ bond by 2.1 (3) ° only. In the anion, both –CO_2_ units are almost orthogonal to the bezene ring mean plane [dihedral angles between –CO_2_ plane and benzene ring (r.m.s. deviation 0.018 Å) are 84.3 (4)° and 86.2 (3) °]. In the crystal structure, cations and anions are linked by N_imidazolyl_–H···O hydrogen bonds to generate a chain formation running along [110](Fig. 2).

### Experimental

An aqueous solution of lead nitrate (0.006 g, 0.2 mmol) in water (5 ml) was added to a mixture of 5-amino-2,4,6-tribromidoisophthalic acid (0.056 g, 0.1 mmol) in water (5 ml) and sodium hydroxide (0.2 ml, 0.5 *M*). To this solution was added 2,2'-biimidazole (0.014 g, 0.1 mmol) in DMF (5 ml). The solution was filtered; slow evaporation yielded pale yellow crystals which were collected (30% yield). CH&N elemental analysis. Calc. for C_14_H_10_Br_3_N_5_O_4_: C 30.46, H 1.83, N 12.69%.; Found: C, 30.38; H, 1.91; N, 12.77%.

### Refinement

Carbon-bound H-atoms were placed in calculated positions (C—H 0.93 Å) and were included in the refinement in the riding model approximation, with *U*_iso_(H) set to 1.2*U*_eq_(C).

The imidazolyl and amino H-atoms were located in a difference Fourier map, and were refined with a distance restraint of N–H 0.88±0.01 Å; their temperature factors were refined.

### Crystal data

**Table:**

C_6_H_8_N_4_^2+^·C_8_H_2_Br_3_NO_4_^2^^−^	*Z* = 2
*M**_r_* = 552.00	*F*(000) = 532
Triclinic, *P*1	*D*_x_ = 2.015 Mg m^−^^3^
Hall symbol: -P 1	Mo *K*α radiation, λ = 0.71073 Å
*a* = 9.0525 (10) Å	Cell parameters from 2718 reflections
*b* = 9.2043 (10) Å	θ = 2.4–27.4°
*c* = 11.5252 (12) Å	µ = 6.68 mm^−^^1^
α = 90.262 (1)°	*T* = 293 K
β = 108.332 (1)°	Prism, pale yellow
γ = 93.136 (1)°	0.35 × 0.25 × 0.15 mm
*V* = 909.96 (17) Å^3^	

### Data collection

**Table:**

Bruker SMART APEX diffractometer	4104 independent reflections
Radiation source: fine-focus sealed tube	3129 reflections with *I* > 2σ(*I*)
graphite	*R*_int_ = 0.025
ω scans	θ_max_ = 27.5°, θ_min_ = 1.9°
Absorption correction: multi-scan (*SADABS*; Sheldrick, 1996)	*h* = −11→11
*T*_min_ = 0.203, *T*_max_ = 0.434	*k* = −11→11
8042 measured reflections	*l* = −14→14

### Refinement

**Table:**

Refinement on *F*^2^	Primary atom site location: structure-invariant direct methods
Least-squares matrix: full	Secondary atom site location: difference Fourier map
*R*[*F*^2^ > 2σ(*F*^2^)] = 0.035	Hydrogen site location: inferred from neighbouring sites
*wR*(*F*^2^) = 0.120	H atoms treated by a mixture of independent and constrained refinement
*S* = 1.12	*w* = 1/[σ^2^(*F*_o_^2^) + (0.0618*P*)^2^ + 0.430*P*] where *P* = (*F*_o_^2^ + 2*F*_c_^2^)/3
4104 reflections	(Δ/σ)_max_ = 0.001
259 parameters	Δρ_max_ = 0.81 e Å^−^^3^
6 restraints	Δρ_min_ = −0.56 e Å^−^^3^

### Fractional atomic coordinates and isotropic or equivalent isotropic displacement parameters (Å^2^)

**Table:**

	*x*	*y*	*z*	*U*_iso_*/*U*_eq_	
Br1	0.44985 (5)	0.68067 (5)	0.04955 (4)	0.03399 (14)	
Br2	0.30262 (7)	0.89508 (6)	0.45860 (5)	0.05335 (18)	
Br3	0.80240 (6)	0.52283 (6)	0.52786 (5)	0.05028 (17)	
O1	0.6388 (4)	0.3751 (3)	0.2311 (3)	0.0364 (7)	
O2	0.8128 (4)	0.5529 (3)	0.2201 (3)	0.0424 (8)	
O3	0.1481 (4)	0.8223 (4)	0.1385 (3)	0.0419 (8)	
O4	0.3322 (4)	1.0034 (3)	0.1754 (4)	0.0465 (9)	
N1	0.5757 (6)	0.7119 (6)	0.5998 (4)	0.0536 (12)	
N2	0.7881 (4)	0.1504 (4)	0.2018 (4)	0.0326 (8)	
N3	0.9353 (4)	−0.0118 (4)	0.1680 (4)	0.0356 (9)	
N4	1.1546 (4)	0.2242 (4)	0.1272 (3)	0.0305 (8)	
N5	1.0016 (4)	0.3864 (4)	0.1539 (3)	0.0258 (7)	
C1	0.6952 (5)	0.5045 (5)	0.2451 (4)	0.0300 (9)	
C2	0.6062 (5)	0.6121 (4)	0.2971 (4)	0.0265 (8)	
C3	0.4913 (5)	0.6934 (4)	0.2216 (4)	0.0246 (8)	
C4	0.4030 (5)	0.7833 (4)	0.2686 (4)	0.0276 (9)	
C5	0.4310 (5)	0.7852 (5)	0.3941 (4)	0.0329 (10)	
C6	0.5465 (5)	0.7061 (5)	0.4750 (4)	0.0343 (10)	
C7	0.6352 (5)	0.6244 (4)	0.4223 (4)	0.0293 (9)	
C8	0.2831 (5)	0.8777 (4)	0.1859 (4)	0.0310 (9)	
C9	0.7208 (6)	0.0171 (5)	0.2144 (5)	0.0491 (13)	
H9	0.6293	−0.0004	0.2337	0.059*	
C10	0.8132 (6)	−0.0853 (5)	0.1932 (5)	0.0485 (13)	
H10	0.7967	−0.1857	0.1954	0.058*	
C11	0.9166 (5)	0.1302 (4)	0.1731 (4)	0.0260 (8)	
C12	1.0206 (5)	0.2449 (4)	0.1518 (4)	0.0253 (8)	
C13	1.2192 (5)	0.3590 (5)	0.1138 (4)	0.0343 (10)	
H13	1.3118	0.3773	0.0960	0.041*	
C14	1.1255 (5)	0.4600 (4)	0.1306 (4)	0.0307 (9)	
H14	1.1414	0.5604	0.1273	0.037*	
H2	0.751 (7)	0.232 (4)	0.217 (5)	0.068 (19)*	
H3	1.005 (5)	−0.054 (5)	0.143 (5)	0.054 (16)*	
H4	1.215 (6)	0.151 (5)	0.139 (6)	0.09 (2)*	
H5	0.923 (4)	0.424 (5)	0.171 (4)	0.035 (13)*	
H11	0.519 (6)	0.764 (6)	0.631 (5)	0.07 (2)*	
H12	0.624 (6)	0.640 (4)	0.643 (4)	0.056 (18)*	

### Atomic displacement parameters (Å^2^)

**Table:**

	*U*^11^	*U*^22^	*U*^33^	*U*^12^	*U*^13^	*U*^23^
Br1	0.0363 (3)	0.0361 (3)	0.0319 (2)	0.01227 (19)	0.01250 (19)	−0.00142 (18)
Br2	0.0560 (4)	0.0543 (4)	0.0623 (4)	0.0200 (3)	0.0340 (3)	−0.0138 (3)
Br3	0.0441 (3)	0.0611 (4)	0.0420 (3)	0.0224 (3)	0.0049 (2)	0.0071 (2)
O1	0.0342 (17)	0.0232 (15)	0.060 (2)	0.0058 (13)	0.0254 (15)	−0.0047 (14)
O2	0.0372 (19)	0.0282 (17)	0.075 (2)	0.0075 (14)	0.0350 (18)	0.0010 (16)
O3	0.0223 (16)	0.0374 (19)	0.063 (2)	0.0091 (14)	0.0084 (15)	−0.0072 (16)
O4	0.0372 (19)	0.0170 (16)	0.088 (3)	0.0121 (14)	0.0217 (18)	0.0094 (16)
N1	0.067 (3)	0.062 (3)	0.036 (2)	0.020 (3)	0.019 (2)	−0.005 (2)
N2	0.0230 (18)	0.0250 (19)	0.055 (2)	0.0067 (15)	0.0198 (17)	−0.0002 (17)
N3	0.028 (2)	0.0250 (19)	0.059 (2)	0.0073 (16)	0.0192 (18)	−0.0025 (17)
N4	0.0188 (17)	0.034 (2)	0.042 (2)	0.0098 (15)	0.0134 (15)	0.0030 (16)
N5	0.0170 (16)	0.0258 (18)	0.0388 (19)	0.0093 (14)	0.0134 (14)	0.0022 (15)
C1	0.034 (2)	0.030 (2)	0.030 (2)	0.0114 (19)	0.0135 (18)	0.0013 (17)
C2	0.027 (2)	0.0187 (19)	0.036 (2)	0.0059 (16)	0.0125 (17)	−0.0015 (16)
C3	0.028 (2)	0.020 (2)	0.0280 (19)	0.0052 (16)	0.0116 (17)	−0.0030 (15)
C4	0.024 (2)	0.021 (2)	0.041 (2)	0.0066 (16)	0.0145 (18)	−0.0016 (17)
C5	0.031 (2)	0.030 (2)	0.044 (2)	0.0102 (19)	0.019 (2)	−0.0103 (19)
C6	0.034 (2)	0.036 (2)	0.036 (2)	0.005 (2)	0.015 (2)	−0.0061 (19)
C7	0.025 (2)	0.024 (2)	0.039 (2)	0.0078 (17)	0.0091 (18)	0.0007 (17)
C8	0.031 (2)	0.020 (2)	0.048 (3)	0.0161 (18)	0.018 (2)	−0.0008 (18)
C9	0.041 (3)	0.034 (3)	0.084 (4)	−0.002 (2)	0.035 (3)	−0.002 (3)
C10	0.042 (3)	0.023 (2)	0.088 (4)	0.002 (2)	0.031 (3)	0.003 (2)
C11	0.029 (2)	0.0151 (19)	0.034 (2)	0.0067 (16)	0.0089 (17)	−0.0003 (16)
C12	0.030 (2)	0.0144 (18)	0.033 (2)	0.0075 (16)	0.0107 (17)	0.0003 (15)
C13	0.036 (2)	0.027 (2)	0.045 (3)	0.0033 (19)	0.019 (2)	0.0031 (19)
C14	0.035 (2)	0.0154 (19)	0.042 (2)	0.0045 (17)	0.0125 (19)	0.0054 (17)

### Geometric parameters (Å, °)

**Table:**

Br1—C3	1.901 (4)	N5—C12	1.325 (5)
Br2—C5	1.895 (4)	N5—C14	1.377 (5)
Br3—C7	1.911 (4)	N5—H5	0.882 (10)
O1—C1	1.259 (5)	C1—C2	1.541 (5)
O2—C1	1.248 (5)	C2—C7	1.386 (6)
O3—C8	1.250 (5)	C2—C3	1.386 (5)
O4—C8	1.237 (5)	C3—C4	1.399 (5)
N1—C6	1.378 (6)	C4—C5	1.388 (6)
N1—H11	0.877 (10)	C4—C8	1.517 (6)
N1—H12	0.879 (10)	C5—C6	1.403 (6)
N2—C11	1.327 (5)	C6—C7	1.396 (6)
N2—C9	1.369 (6)	C9—C10	1.364 (7)
N2—H2	0.880 (10)	C9—H9	0.9300
N3—C11	1.330 (5)	C10—H10	0.9300
N3—C10	1.373 (6)	C11—C12	1.449 (6)
N3—H3	0.879 (10)	C13—C14	1.346 (6)
N4—C12	1.352 (5)	C13—H13	0.9300
N4—C13	1.373 (6)	C14—H14	0.9300
N4—H4	0.880 (10)		
			
C6—N1—H11	120 (4)	C6—C5—Br2	118.5 (3)
C6—N1—H12	118 (4)	N1—C6—C7	121.3 (4)
H11—N1—H12	119 (6)	N1—C6—C5	122.5 (4)
C11—N2—C9	108.4 (4)	C7—C6—C5	116.1 (4)
C11—N2—H2	129 (4)	C2—C7—C6	123.0 (4)
C9—N2—H2	122 (4)	C2—C7—Br3	118.6 (3)
C11—N3—C10	108.3 (4)	C6—C7—Br3	118.4 (3)
C11—N3—H3	127 (4)	O4—C8—O3	127.7 (4)
C10—N3—H3	124 (4)	O4—C8—C4	114.7 (4)
C12—N4—C13	107.4 (4)	O3—C8—C4	117.7 (4)
C12—N4—H4	132 (4)	C10—C9—N2	107.2 (4)
C13—N4—H4	117 (4)	C10—C9—H9	126.4
C12—N5—C14	108.8 (3)	N2—C9—H9	126.4
C12—N5—H5	124 (3)	C9—C10—N3	106.9 (4)
C14—N5—H5	127 (3)	C9—C10—H10	126.6
O2—C1—O1	126.7 (4)	N3—C10—H10	126.6
O2—C1—C2	117.9 (4)	N2—C11—N3	109.2 (4)
O1—C1—C2	115.4 (4)	N2—C11—C12	125.3 (4)
C7—C2—C3	118.2 (4)	N3—C11—C12	125.5 (4)
C7—C2—C1	119.9 (4)	N5—C12—N4	108.7 (3)
C3—C2—C1	121.8 (4)	N5—C12—C11	126.1 (4)
C2—C3—C4	121.7 (4)	N4—C12—C11	125.2 (4)
C2—C3—Br1	119.4 (3)	C14—C13—N4	108.2 (4)
C4—C3—Br1	118.9 (3)	C14—C13—H13	125.9
C5—C4—C3	117.6 (4)	N4—C13—H13	125.9
C5—C4—C8	121.0 (3)	C13—C14—N5	106.9 (4)
C3—C4—C8	121.3 (4)	C13—C14—H14	126.5
C4—C5—C6	123.2 (4)	N5—C14—H14	126.5
C4—C5—Br2	118.3 (3)		
			
O2—C1—C2—C7	−96.8 (5)	C5—C6—C7—C2	−4.4 (6)
O1—C1—C2—C7	83.9 (5)	N1—C6—C7—Br3	−1.2 (6)
O2—C1—C2—C3	86.3 (5)	C5—C6—C7—Br3	176.5 (3)
O1—C1—C2—C3	−93.0 (5)	C5—C4—C8—O4	85.3 (5)
C7—C2—C3—C4	−1.5 (6)	C3—C4—C8—O4	−93.6 (5)
C1—C2—C3—C4	175.4 (4)	C5—C4—C8—O3	−93.5 (5)
C7—C2—C3—Br1	179.4 (3)	C3—C4—C8—O3	87.6 (5)
C1—C2—C3—Br1	−3.7 (5)	C11—N2—C9—C10	0.4 (6)
C2—C3—C4—C5	−2.0 (6)	N2—C9—C10—N3	0.0 (7)
Br1—C3—C4—C5	177.1 (3)	C11—N3—C10—C9	−0.4 (6)
C2—C3—C4—C8	176.9 (4)	C9—N2—C11—N3	−0.7 (5)
Br1—C3—C4—C8	−4.1 (5)	C9—N2—C11—C12	179.9 (4)
C3—C4—C5—C6	2.5 (6)	C10—N3—C11—N2	0.7 (5)
C8—C4—C5—C6	−176.4 (4)	C10—N3—C11—C12	−179.9 (4)
C3—C4—C5—Br2	−175.5 (3)	C14—N5—C12—N4	−0.2 (5)
C8—C4—C5—Br2	5.6 (6)	C14—N5—C12—C11	179.4 (4)
C4—C5—C6—N1	178.2 (5)	C13—N4—C12—N5	0.0 (5)
Br2—C5—C6—N1	−3.8 (6)	C13—N4—C12—C11	−179.7 (4)
C4—C5—C6—C7	0.6 (7)	N2—C11—C12—N5	−2.3 (7)
Br2—C5—C6—C7	178.6 (3)	N3—C11—C12—N5	178.4 (4)
C3—C2—C7—C6	4.9 (6)	N2—C11—C12—N4	177.3 (4)
C1—C2—C7—C6	−172.0 (4)	N3—C11—C12—N4	−2.0 (7)
C3—C2—C7—Br3	−176.0 (3)	C12—N4—C13—C14	0.2 (5)
C1—C2—C7—Br3	7.0 (5)	N4—C13—C14—N5	−0.4 (5)
N1—C6—C7—C2	177.9 (4)	C12—N5—C14—C13	0.4 (5)

### Hydrogen-bond geometry (Å, °)

**Table:**

*D*—H···*A*	*D*—H	H···*A*	*D*···*A*	*D*—H···*A*
N2—H2···O1	0.88 (1)	1.74 (2)	2.608 (4)	168 (6)
N3—H3···O3^i^	0.88 (1)	1.78 (2)	2.624 (5)	160 (5)
N4—H4···O4^i^	0.88 (1)	1.74 (1)	2.614 (5)	175 (7)
N5—H5···O2	0.88 (1)	1.79 (2)	2.636 (4)	160 (4)

Symmetry codes: (i) *x*+1, *y*−1, *z*.
